# Does kynurenic acid act on nicotinic receptors? An assessment of the evidence

**DOI:** 10.1111/jnc.14907

**Published:** 2019-11-24

**Authors:** Trevor W. Stone

**Affiliations:** ^1^ Institute for Neuroscience and Psychology University of Glasgow Glasgow G12 8QQ UK; ^2^ Present address: Kennedy Institute NDORMS University of Oxford Oxford OX3 7FY UK

**Keywords:** cholinergic receptors, galantamine, kynurenic acid, kynurenine, nicotinic receptors, NMDA receptors

## Abstract

As a major metabolite of kynurenine in the oxidative metabolism of tryptophan, kynurenic acid is of considerable biological and clinical importance as an endogenous antagonist of glutamate in the central nervous system. It is most active as an antagonist at receptors sensitive to N‐methyl‐D‐aspartate (NMDA) which regulate neuronal excitability and plasticity, brain development and behaviour. It is also thought to play a causative role in hypo‐glutamatergic conditions such as schizophrenia, and a protective role in several neurodegenerative disorders, notably Huntington’s disease. An additional hypothesis, that kynurenic acid could block nicotinic receptors for acetylcholine in the central nervous system has been proposed as an alternative mechanism of action of kynurenate. However, the evidence for this alternative mechanism is highly controversial, partly because at least eight earlier studies concluded that kynurenic acid blocked NMDA receptors but not nicotinic receptors and five subsequent, independent studies designed to repeat the results have failed to do so. Many studies considered to support the alternative ‘nicotinic’ hypothesis have been based on the use of analogs of kynurenate such as 7‐chloro‐kynurenic acid, or putatively nicotinic modulators such as galantamine, but a detailed analysis of the pharmacology of these compounds suggests that the results have often been misinterpreted, especially since the pharmacology of galantamine itself has been disputed. This review examines the evidence in detail, with the conclusion that there is no confirmed, reliable evidence for an antagonist activity of kynurenic acid at nicotinic receptors. Therefore, since there is overwhelming evidence for kynurenate acting at ionotropic glutamate receptors, especially NMDAR glutamate and glycine sites, with some activity at GPR35 sites and Aryl Hydrocarbon Receptors, results with kynurenic acid should be interpreted only in terms of these confirmed sites of action.

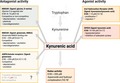

## Graphical abstract

Kynurenic acid has well‐established activity as an antagonist at glutamate receptors, especially the glutamate and glycine co‐agonist binding sites, with possibly relevant actions on Aryl Hydrocarbon Receptors and the GPR35 binding site. A single proposal that it also acts on acetylcholine nicotinic receptors is analysed and discussed in the light of at least 12 failures to reproduce the activity, and a wide range of pharmacological arguments. It is concluded that there is no confirmed, reliable evidence for any effect at nicotinic acetylcholine receptors.

**Abbreviations used**
2‐AP5: 2‐amino‐5‐phosphono‐pentanoic acid
57diCKA: 5,7‐dichlorokynurenic acid
5HT: 5‐hydroxytryptamine
7CK: 7‐chlorokynurenic acid
AHR: aryl hydrocarbon receptor
AMPA: α‐amino‐3‐hydroxy‐5‐methyl‐4‐isoxazole‐(
APL: allosteric potentiating https://en.wikipedia.org/wiki/Ligand_(biochemistry)
CHO: chinese hamster ovary cells
CNS: central nervous system
DhβE: dihydro‐β‐erythroidine
DMSO: dimethyl‐sulfoxide
EPSC: excitatory postsynaptic currents
EPSP: excitatory postsynaptic potentials
GABA: γ‐amino‐butyric acid
GPR35: G‐protein‐coupled receptor‐35
IDO: indoleamine‐2,3‐dioxygenases
IPSC: inhibitory post‐synaptic current
IPSP: inhibitory post‐synaptic potential
KAT: kynurenine aminotransferase
KMO: kynurenine‐3‐monoxygenase
LTP: long‐term potentiation
MK‐801: dizocilpine
MLA: methyl‐lycaconitine
NAD: nicotinamide adenine dinucleotide
NMDA: N‐methyl‐D‐aspartic acid
NR1/2/3: NMDA receptor subunit
PSP: post‐synaptic potential
SDZ‐220‐581: ((S)‐α‐amino‐2′‐chloro‐5‐(phosphonomethyl)[1,1′‐biphenyl]‐3‐propanoic acid)
TDO: tryptophan‐2,3‐dioxygenase

## Introduction: the kynurenine pathway and receptors

**Figure 1:**
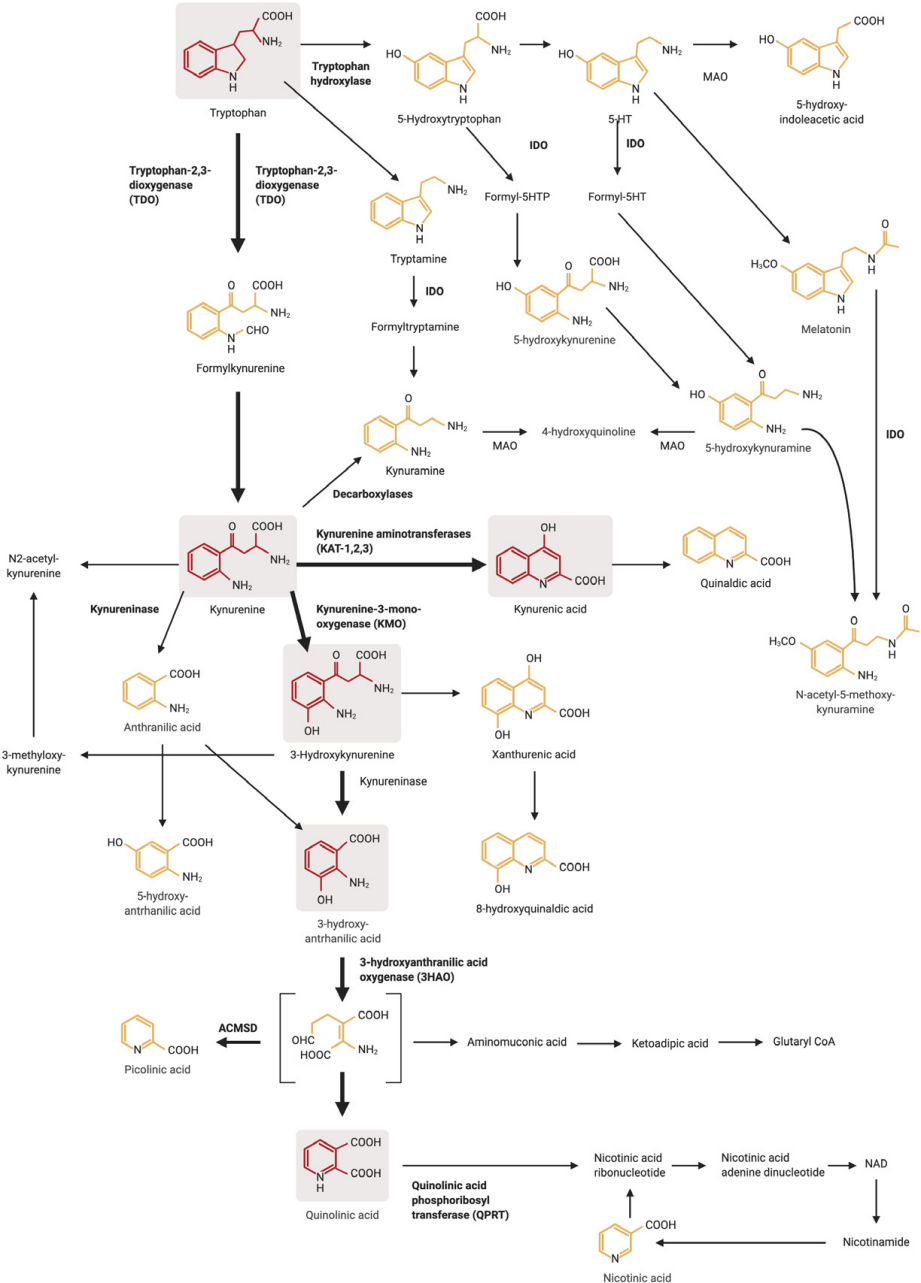
A schematic of the kynurenine pathway of tryptophan metabolism.

**Figure 2:**
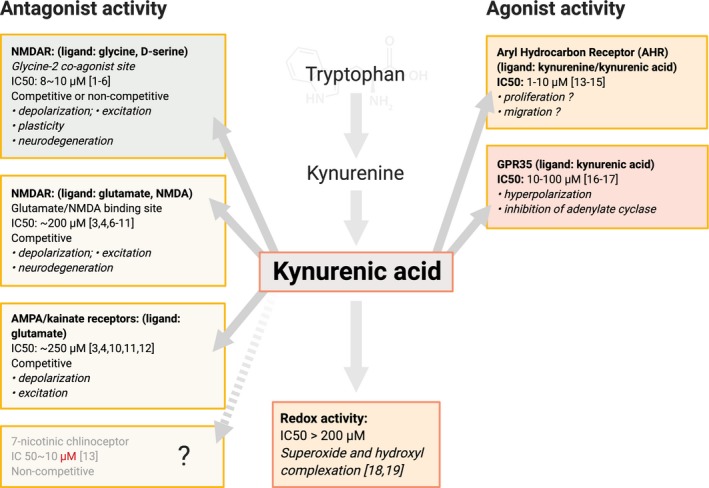
A summary of the major molecular targets known for kynurenic acid, with an indication of the qualitative and quantitative activity expanded in the text. The citation numbers refer to the following: [1] Henderson et al., 1990; [2] Watson et al. 1988; [3] Kessler et al. 1989a; [4] Kessler et al. 1989b; [5] Kloog et al. 1990; [6] Mayer et al. 1988; [7] Olverman et al. 1988; [8] Moroni et al. 1989; [9] Robinson et al. 1985; [10] Danysz et al. 1989a; [11] Danysz et al. 1989b; [12] Fisher and Mott, 2011; [13] DiNatale et al. 2010; [14] Opitz et al. 2011; [15] Kimura et al. 2017; [16] Wang et al. 2006; [17] Guo et al. 2008; [18] Kubicova et al. 2019; [19] Lugo‐Huitron et al. 2011.

The kynurenine pathway accounts for the metabolism of around 95% of free tryptophan (Fig. 1). Kynurenine is a product of tryptophan oxidation by indoleamine‐2,3‐dioxygenases (IDO‐1 or IDO‐2) or tryptophan‐2,3‐dioxygenase (TDO) and is further oxidised to quinolinic acid and nicotinamide adenine dinucleotide. Interest in the pharmacological importance of the kynurenine pathway in the central nervous system (CNS) dates from the observation that quinolinic acid is a selective agonist at ionotropic glutamate receptors sensitive to N‐methyl‐D‐aspartate (NMDA), producing neuronal excitation (Stone and Perkins 1981; Perkins and Stone, 1983). Shortly afterwards it was found that another tryptophan catabolite, kynurenic acid, also had pharmacological activity, blocking glutamate receptors especially those activated by NMDA (Perkins and Stone 1982). The conclusion that kynurenate was a selective antagonist was supported by a subsequent series of functional studies detailed below, and by Sakurai *et al. *(1991) who showed that the highly selective NMDA receptor blocker dizocilpine (MK‐801) was displaced by kynurenic acid, while others have shown a lack of activity at a range of receptor sites including, most recently, opiate binding (Zador *et al.* 2014) (Fig. 2).

Since then evidence has been obtained for a major role of NMDA receptors in synaptic transmission and plasticity including learning and memory and other aspects of cognition (Tsien *et al.* 1996; Cammarota *et al.* 2000; Bartlett *et al.* 2007; Berberich *et al.* 2007; Zorumski and Izumi 2012; Paoletti *et al.* 2013; Mukherjee *et al.* 2014). Since maintained or excessive activation of NMDA receptors causes cellular calcium overload, quinolinic acid can produce excitotoxicity and neurodegeneration (Stone and Addae 2002; Kalia *et al.* 2008; Hardingham 2009; Hardingham and Bading 2010; Danysz and Parsons, 2012; Guillemin, 2012; Stone *et al.* 2012a,b; Lovelace *et al.* 2017), again balanced or prevented by endogenous levels of kynurenic acid (Harris *et al.* 1998; Sapko *et al.* 2006; Stone and Darlington 2013). This balance between quinolinic acid as an agonist and kynurenic acid as an antagonist can be controlled by the relative expression and activity of kynurenine aminotransferases (KATs), kynureninase and kynurenine‐3‐mono‐oxygenase (Fig. 1), perhaps indicating a significant physiological or pathological relevance of the quinolinate/kynurenate ratio. Correspondingly, disorders of cognition have been linked with high levels of kynurenate and ascribed to the slowing or suppression of excitatory neurotransmission by this compound (Stone and Darlington 2013; Schwieler *et al.* 2015; Erhardt *et al.* 2017).

Schizophrenia, for example, has been linked closely with kynurenic acid concentrations in the CNS (Stone and Darlington 2013; Cho *et al.* 2017; Plitman *et al.* 2017; Erhardt *et al.* 2017; Ogyu *et al.* 2018; Laumet *et al.* 2017; Dantzer *et al.* 2011; Kindler *et al.* 2019). In schizophrenia, the concentration of kynurenate in the brain is elevated above normal and nucleotide polymorphisms of kynurenine pathway enzymes have been identified (Wonodi and Schwarcz 2010; Holtze *et al.* 2012; Stone and Darlington 2013; Erhardt *et al.* 2017; Hahn *et al.* 2018). Work in progress is attempting to identify inhibitors of KAT as potential treatments for schizophrenia. Conversely, raising kynurenic acid levels in the CNS by inhibiting the enzyme kynurenine‐3‐monoxygenase (Forrest *et al.* 2016) has overall neuro‐inhibitory and neuroprotective effects which are potentially of value in the treatment of neurodegenerative disorders, especially Huntington's disease (Stone 2001a; Stoy *et al.* 2005; Giorgini *et al.,* 2005, 2013; Guidetti *et al.* 2006; Forrest *et al.* 2010; Schwarcz *et al.* 2010; Stone *et al.* 2012a,b; Lovelace *et al.* 2017; Jacobs *et al.,* 2018). Kynurenine metabolites, especially quinolinic acid and kynurenic acid may also be involved in the brain damage resulting from cerebral infarcts (strokes), and may be involved in dysfunctional conditions such as multiple sclerosis (Lovelace *et al.* 2016, 2017; Lim *et al.* 2017).

Perhaps even more importantly, raising kynurenic acid levels in the developing embryo or neonatal animals results in changes in CNS anatomy (neocortex, hippocampus, cerebellum), physiology and behaviour which persist well into adulthood (Pocivavsek *et al.* 2012; Alexander *et al.,* 2012, 2013; Forrest *et al.,* 2013a,b; Khalil *et al.,* 2014; Pisar *et al.* 2014). This carries fundamental implications for the effects of environmental influences on embryonic development in humans, since infections and inflammation induce and activate IDO‐1 and other enzymes in the kynurenine pathway, while stress – via the hypothalamo‐pituitary‐adrenal axis and the secretion of corticosteroids – induces and activates tryptophan‐2,3‐dioxygenase (Fatokun *et al.* 2013).

In view of these clinical connections it is important to understand the sites and mechanisms of action of kynurenic acid (Schwarcz and Stone 2017). The blockade of NMDA receptors has been confirmed and supported in countless reports since 1982 as detailed below, with kynurenate rapidly becoming a routine tool for the exploration of glutamate’s physiological activity and pathological relevance (see Stone and Burton 1988; Stone 1993; Stone and Darlington, 2002; Moroni *et al.* 2012; Schwarcz *et al.* 2012). In addition, a large number of compounds developed to block glutamate activity are based on the structure of kynurenic acid (see Stone 2000a,b), including halogenated derivatives (Kemp *et al* 1988; Foster *et al* 1992), quinoxalines (Leeson *et al.* 1991; Leeson and Iversen, 1994), tetrahydroquinolines (Carling *et al.* 1992; Leeson *et al.* 1992; Cai *et al.* 1996) and indolyl‐propionic acid derivatives (Salituro *et al.* 1992). The status and value of kynurenate as an endogenous, reliable and reproducible antagonist at glutamate receptors, especially NMDA receptors is, therefore, very well established from functional, binding and electrophysiological analyses as detailed in the following sections.

Throughout most of this earlier work the selectivity of kynurenate and its interactions with glutamatergic systems was emphasised by the *absence* of any interaction with cholinergic receptors, as will be described below. A debate has developed, however, initiated by a report that kynurenic acid could block nicotinic receptors for acetylcholine and other ligands (Hilmas *et al.* 2001), possibly at lower concentrations than those which blocked NMDAR. This concept has been used to interpret a range of results using kynurenate and its synthetic analogs and to argue that the primary physiological and pathological role of kynurenic acid may be mediated by a blockade of nicotinic receptors in the CNS. The present overview examines the evidence for actions of kynurenic acid on NMDA and nicotinic receptors and assesses the validity of studies which have been based on these concepts.

## Functional studies on kynurenic acid

### Electrophysiological studies

In the first studies to report receptor‐specific actions of compounds in the kynurenine pathway, quinolinic acid was found to depolarise neurons in the cerebral cortex of anaesthetised rats (Stone and Perkins, 1981, Perkins and Stone, 1983), and actions mediated by NMDA receptors since it was blocked selectively by the newly reported and highly selective antagonists 2‐amino‐5‐phosphono‐pentanoic acid (Davies *et al.* 1981; Perkins *et al.* 1981; Stone *et al.* 1981) and its more potent analogue 2‐amino‐7‐phosphono‐heptanoic acid (Perkins *et al.* 1982; Stone 1986). Subsequent testing of other compounds in the kynurenine pathway using similar microiontophoretic applications to single neurons *in vivo* (Stone 1985) led to the identification of the antagonistic activity of kynurenic acid (Perkins and Stone 1982). Kynurenate was found to block all three ionotropic receptor subtypes for glutamic acid (known then as NMDA, kainate and quisqualate receptors) but with greatest potency blocking NMDA receptors (Perkins and Stone 1982). This receptor selectivity of kynurenate was later confirmed in different regions of the CNS (Ganong *et al.* 1983; Elmslie and Yoshikami 1985; Herrling 1985; Peet *et al.,* 1986; Cotman *et al.,* 1986; Curry *et al.,* 1986; Ganong and Cotman, 1986), although the potency of kynurenate as antagonist varied, being active at concentrations fivefold lower in the spinal cord, for example, than in the neocortex (Ganong *et al* 1983).

Correspondingly, kynurenate was soon found to inhibit monosynaptic and polysynaptic potentials in the spinal cord and hippocampus (Ganong *et al.,* 1983; Stone and Perkins, 1984; Harris and Cotman, 1985; Stevens and Cotman, 1986) and to inhibit spontaneous miniature glutamate‐mediated synaptic potentials (Cotman *et al.* 1986). The postsynaptic site of action of kynurenate was confirmed in a quantal analysis of synaptic transmission which revealed a decrease in the mean quantal size of excitatory potentials by kynurenate, but no change in the number of quanta released per stimulus (Brooks *et al.* 1986), consistent with an action of kynurenate at postsynaptic targets.

Results from these studies also showed that kynurenic acid had no effect on the basic biophysical properties of neurons (such as resting membrane potential, input resistance, or action potential thresholds) even at concentrations of kynurenate higher than those acting on amino acid receptors, thus eliminating non‐specific actions on cell polarisation or excitability (Ganong *et al.,* 1983; Herrling, 1985; Jahr and Jessell, 1985; Ganong and Cotman, 1986; Brady and Swann, 1988; Cherubini *et al.,* 1988a,b; Lewis *et al.,* 1989).

In several of these early studies, acetylcholine, nicotine or a nicotinic agonist were included as control for the amino acid selectivity of kynurenate. Concentrations of kynurenate sufficient to block glutamate responses and synaptic potentials had no effect on responses to acetylcholine (Perkins and Stone 1982; Jahr and Jessell 1985; Jahr and Yoshioka, 1986; Tsumoto *et al.,* 1986; Perrins and Roberts, 1994), broad spectrum nicotinic agonists such as carbachol (Jahr and Yoshioka,^1986^; Bijak *et al.* 1991) or nicotine itself (Perrins and Roberts, 1994; Bertolino *et al* 1997) which could be blocked by mecamylamine but not by kynurenic acid even at millimolar concentrations. Also at 1 mM, kynurenic acid had no effect on nicotinic excitatory postsynaptic potentials (EPSPs) and was used to help distinguish these from glutamate‐mediated EPSPs (Perrins and Roberts, 1995) (Fig. 2).

Despite these various reports that kynurenic acid did *not* affect nicotinic receptors, it was later claimed reported that kynurenic acid could block α7‐nicotinic receptors expressed in cultured hippocampal or neocortical neurons and hippocampal slices. Hilmas *et al. *(2001) Choline was used as a nicotinic receptor agonist to induce whole‐cell inhibitory post‐synaptic currents (IPSCs) which were reduced by kynurenic acid. The results were interpreted as choline acting on nicotinic receptors on γ‐amino‐butyric acid (GABA)‐releasing neurons, either depolarising the cell bodies to initiate the invasion of GABA‐releasing terminals by action potentials, or by acting on cholinoceptors located on synaptic terminals and able to induce GABA release directly (independently of action potentials). Kynurenic acid was then assumed to block those cholinoceptors (somatic or terminal), reducing the choline‐induced increase in frequency of GABA‐mediated IPSCs. This result contrasts with earlier and subsequent studies (above and Table 1) in which kynurenate had no effect on nicotinic receptor activity.

**Table 1:** A summary of studies testing kynurenic acid against nicotinic receptor responses

Experimental preparation	Summary of relevant results	References
Anesthetised rat; single neuron responses in cerebral neocortex	Kynurenic acid:‐ ‐ blocked responses to NMDA and quisqualate ‐ little effect on responses to acetylcholine	Perkins and Stone, (1982)
Synaptic potentials in hemisected spinal cord	Kynurenic acid:‐ ‐ blocked responses to NMDA, glutamate ‐ no effect (2.5 mM) on cholinergic EPSPs	Elmslie and Yoshikami, (1985)
*Xenopus* spinal neurons	Kynurenic acid:‐ ‐ blocked glutamate‐mediated EPSPs ‐ no effect on nicotinic receptor‐mediated EPSPs	Perrins & Roberts (1984)
Dorsal root ganglion cell synapses onto dorsal horn spinal neurons	Kynurenic acid:‐ ‐ blocked excitatory amino acid responses and EPSPs ‐ no effect on ATP‐induced depolarisation	Jahr and Jessell, (1985)
Rat spinal cord *in vitro*	Kynurenic acid:‐ ‐ blocked glutamate‐mediated EPSPs and responses to glutamate or NMDA ‐ no effect on carbachol‐induced depolarisation	Jahr and Yoshioka 1986
Cat visual cortex neurons *in vivo*	Kynurenic acid:‐ ‐ blocked glutamate and aspartate responses ‐ no effect on acetylcholine‐induced excitation	Tsumoto *et al. *(1986)
Dissociated midbrain neurons in culture	Kynurenic acid:‐ ‐ blocked EPSCs and responses to quisqualate and AMPA ‐ no effect on excitation induced by acetylcholine or nicotine	Bijak *et al. *(1991)
Neurons in the rat dorsal motor nucleus of the vagus *in vitro*	Kynurenic acid: ‐ ‐ no effect (1 mM) on excitatory activity of nicotine or nicotinic agonists epibatidine and cytisine	Bertolino *et al. * 1997
Cultured hippocampal neurons	Kynurenic acid: ‐ ‐ blocked NMDA‐mediated excitation (IC50 = 15μM) ‐ blocked nicotinic depolarization (IC50 = 7μM)	Hilmas *et al. *(2001)
Rat neocortex slices	Kynurenic acid:‐ ‐ does not block (5 mM) nicotinic EPSPs in neocortex	Chu et al. (2000)
Interneurons in rat hippocampal slices *in vitro*	Kynurenic acid:‐ ‐ possible reduction of small, putatively nicotinic EPSPs in complex receptor and channel blocking medium	Stone, (2007)
Primary hippocampal neurons in culture	Kynurenic acid:‐ ‐ no blockade (1 mM) of nicotinic excitation	Arnaiz‐Cot *et al. *(2008)
Human cell lines (HEK293‐MSRII and α7NR‐expressing GH4‐α7), transfected with human or rat NMDAR subunits; cultured primary neurons	Kynurenic acid:‐ ‐ no effect (up to 3 mM) on responses to acetylcholine or nicotinic agonists	Mok *et al. *(2009)
Interneurons in rat hippocampal slices	Kynurenic acid:‐ ‐ blocked glutamate‐mediated EPSCs ‐ no effect on responses to nicotinic receptor activation	Dobelis *et al. *(2012)
The following studies examined kynurenic acid on general neuronal properties		
Various	Kynurenic acid:‐ ‐ No effect on membrane potentials or resistance, indicating no non‐specific depression of excitability or membrane function	Ganong *et al., *(1983); Herrling, (1985); Jahr and Jessell, (1985); Ganong and Cotman, (1986); Brady and Swann, (1988); Cherubini *et al., *(1988a,b); Lewis *et al., *(1989)

In cultured cells kynurenic acid was reported to be more potent than on slices, with nanomolar concentrations being sufficient to inhibit significantly the responses to choline (Hilmas *et al.* 2001). The difference between cultures and slices was attributed to the absence of ‘diffusion barriers’ in the former which might impede the access of kynurenate to sites of action in intact, freshly prepared slices. While this is probably true, the low nanomolar potency of kynurenate has become widely cited in relation to work not only on cell cultures but also in slices and *in vivo*, where it is now often assumed that kynurenate would be as active as in culture. Even if the results are correct, the concentrations of kynurenate claimed to reduce α7‐nicotinic receptor responses exhibit a very shallow concentration‐response relationship: although nanonolar levels were reported to have minimal activity on nicotinic responses, concentrations of around 100 μM were required to inhibit choline responses by ~60% (90% inhibition at 1 mM). These activities are in the same micromolar range which block NMDA receptors in brain slices examined in previous studies. In the brain slice experiments of Hilmas *et al. *(2001) kynurenate at 1 mM reduced choline responses by 50–75%, a smaller inhibition than is normally obtained against NMDA.

Since the results of these studies contradict all of the earlier work outlined above which could find no effect of kynurenic acid on nicotinic receptors, there is a need to re‐evaluate the results and interpretation of the work in comparison with related work from other laboratories.

### Assessing reproducibility

Since the original work had been based primarily on the use of cell cultures, one study was performed to examine the possibility of nicotinic receptor antagonism in more detail in intact hippocampal slices, using single cell recordings from interneurons in the stratum radiatum (Stone 2007). When tested initially for its effects on depolarising actions of NMDARs and nicotinic receptors, kynurenic acid blocked the former at micromolar concentrations but had no effect on NMDARs or nicotinic receptors when applied at submicromolar levels. This failure to observe any high potency effects of kynurenate might reflect the major differences between intact tissues and cultured cells where the use of artificial media, the loss of pericellular barriers, changes in intercellular adhesion, the absence of natural ligands for receptors and a lack of critical compounds or factors found in tissues (enzymes, inhibitors, growth factors, hormones, trace elements etc), may affect the physiological status of the cells and their sensitivity to test compounds. It was therefore considered that the different results of Hilmas *et al *(2001) and Stone (2007) might be attributable to these various technical differences, and the work was extended to examine the effects of kynurenate on synaptic transmission, testing bath‐applied kynurenic acid at known concentrations on α7‐nicotinic receptor‐mediated EPSPs.

α7‐nicotinic receptors mediate fast cholinergic transmission onto hippocampal interneurons and EPSPs are blocked by the selective nicotinic antagonist methyl‐lycaconitine (MLA) (Alkondon *et al.* 1992; Stone 2007). These EPSPs are difficult to detect and a cocktail of antagonists was used to isolate them by blocking muscarinic, GABA‐A, GABA‐B, 5‐hydroxytryptamine‐3 (5‐HT3), α‐amino‐3‐hydroxy‐5‐methyl‐4‐isoxazole‐propionic acid (AMPA) and NMDA receptors to suppress non‐nicotinic transmission (Stone 2007). Even excluding these various synaptic influences on EPSP generation, nicotinic EPSPs can be demonstrated on only a few neurons and they are usually very small relative to the residual background activity.

Under these restrictive conditions, kynurenic acid did appear to reduce EPSP size but several important factors must be emphasised. Firstly and most importantly, the concentrations of kynurenic acid needed to reduce nicotinic transmission (EC50 136μM), were similar to those which block NMDA receptors – similar to those reported by Hilmas *et al. *(2001) in slices but orders of magnitude greater than those claimed to work in cell cultures (Hilmas *et al.* 2001).

Secondly, the reduction in EPSP size was *consistent* with α7‐nicotinic receptor blockade, but does not represent proof since the apparent blockade could have been indirect, or a result of reduced acetylcholine release (see below).

Thirdly, the cocktail of ionotropic receptor blocking agents listed above, used at relatively high concentrations to maximise the blockade of interfering receptors needed to isolate nicotinic EPSPs, could have affected the nicotinic ionotropic receptors or ion channels associated with excitability and transmitter release in an isolated, vulnerable preparation. The mixture of antagonists could also have modified functional receptor interactions which might make cells more susceptible to kynurenic acid. Atropine, for example, has been found to have some activity on nicotinic receptors (Gonzalez‐Rubio *et al.* 2006). It is also likely that the antagonist cocktail did not totally block excitatory sensitivity to all the amino acids, neuropeptides (such as neurokinins, cholecystokinin, neuropeptide Y, opioid peptides), or other endogenous neuroactive compounds which affect neuronal excitability. These results clearly demonstrated a total absence of kynurenate blockade of nicotinic receptors at low concentrations with possibly weak activity at high micromolar levels when it was difficult to exclude effects on other excitatory mediators.

Lastly, the potential actions of kynurenic acid on the Aryl Hydrocarbon Receptors (Opitz *et al.* 2011) or the orphan G‐protein‐coupled receptor G‐protein‐coupled receptor‐35 (GPR35) (Wang *et al.* 2006; Mackenzie and Milligan 2015; Shore and Reggio, 2015) were not considered by Stone (2007) since kynurenines had not yet been linked with their activity. AHRs have received little attention in the CNS since primary interest has been in their role in immune system cells and the regulation of kynurenine pathway activation in tolerogenesis. Neverthelesss, they play an important role in the kynurenine‐ AHR‐IDO cycle which is a key factor in immune tolerogenesis and tumour development, where they are sensitive to physiological concentrations of kynurenine and kynurenic acid (Opitz *et al.* 2011; Bessede *et al.* 2014). Consistent with this, AHRs can regulate neuronal development, differentiation and synaptic function (Huang *et al.* 2004, 2011; Qin and Powell‐Coffman 2004), neuronal or glial migration (Kimura *et al.* 2017) and may modulate cholinesterase activity and hypoxic sensitivity (Xie *et al.* 2013; Rzemieniec *et al.* 2016). The concentrations of kynurenate which activate AHRs are generally the same or lower than those acting on amino acid receptors (Fig. 2) (DiNatale *et al.* 2010; Opitz *et al.* 2011).

The G‐protein‐coupled receptor GPR35 is expressed in glia and neurons of the hippocampus (Berlinguer‐Palmini *et al.* 2013; Alkondon *et al* 2015). When activated they inhibit adenylate cyclase and depress pain‐induced behaviours (Rojewska et al, 2018; Cosi *et al.* 2011; Moroni 2012; Resta *et al.* 2016). On neurons, they depress excitability and synaptic transmission in the hippocampus (Alkondon *et al.* 2011) and dorsal root ganglia, where they depress N‐type calcium channels (Guo *et al.,* 2008; Ohshiro *et al.* 2008) possibly mediated by Hyperpolarisation‐activated‐Cyclic Nucleotide‐gated ion channels (Resta *et al.* 2016). In general, concentrations of kynurenate required to activate GPR35 are of the same order as those which block NMDA receptors (Wang *et al.* 2006; Berlinguer‐Palmini *et al.* 2013). In Chinese Hamster Ovary cells expressing GPR35, kynurenic acid was more potent on rat GPR35 (EC50 7.4 μM) than mouse (10.7 μM) or human (39.2) receptors (Wang *et al.* 2006). The latter activity was similar to that observed to reduce calcium currents in rat superior cervical ganglion neurons (EC50 58 μM) (Guo *et al. et al.* 2008). These sites may be physiologically relevant and able to contribute to the effects of kynurenic acid under appropriate conditions.

Later work from Mok *et al. *(2009) examined two human cell lines (HEK293‐MSRII and the α7nicotinic receptor‐expressing GH4‐α7 line). The former were transfected with human or rat NMDAR subunits (NR1A, NR2A and NR2B) and were tested at 37°C after 24–48 h. When tested on the NMDA receptor subunits, kynurenic acid blocked NR1A/NR2A combinations with an IC50 of 24.4 μM (in 1 μM glycine) up to 158 μM (at high 30 μM glycine), entirely consistent with the large majority of earlier studies described above. Similar results were obtained using the transfected rat proteins or when tested on cultured rat hippocampal neurons. However, the group found no effect of kynurenic acid on acetylcholine‐evoked, methyl‐lycaconitine (MLA)‐sensitive current responses to choline or acetylcholine at concentrations up to 3 mM in the three different test systems. To circumvent the uncertainties resulting from highly localised ejections of drugs onto neurons in a complex system such as hippocampal slices, acetylcholine was also administered by microinjection into rat slices, but here again, kynurenic acid at 1 mM had no effect on nicotinic receptor responses.

An important observation in this study was that dimethyl‐sulfoxide (DMSO), used as the solvent for kynurenic acid by Hilmas *et al. *(2001) did reduce α7‐nicotinic receptor sensitivity at the relevant concentration range needed for solubilising kynurenic acid, raising the possibility that DMSO could have been responsible for the results of Hilmas *et al. *(2001). The same conclusion was drawn by Dobelis et al. as discussed below (*Technical issues*).

In addition to these two reports (Stone 2007; Mok *et al.* 2009), two other, independent studies have been published on the effects of kynurenate on nicotinic receptors. Arnaiz‐Cot *et al. *(2008) used cultures of primary, embryonic hippocampal neurons to allow a direct comparison with the earlier work and nicotinic depolarisation was induced by the non‐selective nicotinic receptor agonist choline. Kynurenic acid had no effect on this choline response, even at a concentration of 1 mM.

These results are entirely in accord with those of Mok *et al. *(2009) but they also led the authors to note that α7‐nicotinic receptors are expressed at several sites and on different neuron types. Hippocampal GABA‐releasing interneurons are major sites of nicotinic receptors (Freedman *et al.,* 1993; Adams *et al.,* 2001; Alkondon *et al.* 2004) providing a means by which their activation can regulate the excitability of GABA‐releasing neurons and change the frequency of PSPs in postsynaptic cells (Pitler and Alger 1992; Maggi *et al.,* 2001). This view is one of many consistent with the concept that nicotinic receptor activity is associated primarily with modulating cell sensitivity to other neurotransmitters, as well as, or instead of, mediating classical fast nicotinic synaptic transmission (Gray *et al.,* 1996; Role and Berg, 1996; Bertolino *et al.,* 1997; Ji and Dani, 2000). Other authors have reached similar conclusions, such as that fast neurotransmission rarely involves nicotinic receptor activation in the CNS (Frazier *et al.,* 1998; Alkondon *et al.* 2004; Role and Berg, 1996; see Dani and Bertrand, 2007) or that nicotinic postsynaptic potentials are so small that they are unlikely greatly to affect neural excitability (McQuiston and Madison, 1999). This would also be consistent with indications that most central nicotinic receptors are functionally expressed mainly on presynaptic terminals with their main function to modulate the release of neurotransmitters (Wonnacott, 1997) (see below).

Finally, Dobelis *et al. *(2012) examined the effects of choline applied at intervals from micropipettes via a pressure system (see Stone 1985) to prevent desensitisation, onto interneurons in the stratum radiatum and hilar regions of hippocampal slices from young or adult rats and mice. Voltage clamp analysis indicated that kynurenate would block completely spontaneous glutamate‐mediated excitatory postsynaptic currents without affecting α7‐nicotinic receptor responses to choline. The failure to affect nicotinic receptor responses was reported even in high (1 mM) kynurenate after incubation for 90 min to eliminate the possibility of slow receptor blockade kinetics. This result not only confirms the results of Stone (2007), Arnaiz‐Cot *et al. *(2008) and Mok *et al. *(2009) but indicates that the failure to block α7‐nicotinic receptor responses was not restricted to the stratum radiatum region of the hippocampus. It should be noted also that the failure to observe nicotinic receptor blockade even in cell cultures (Arnaiz‐Cot *et al.* 2008; Mok *et al.* 2009) indicates that the absence of diffusion barriers, and other considerations listed above in such experiments, cannot account for the reported results of Hilmas *et al. *(2001).

### Enzyme deletion

In a follow‐up study to examine the question of kynurenate activity on nicotinic receptors in a more indirect manner, Alkondon *et al. *(2004) and Yu *et al. *(2004) examined the effect of reducing endogenous levels of kynurenic acid by deleting its synthesising enzyme KAT‐2. The reduced kynurenic acid concentrations were associated with an increase in the activity of α7‐nicotinic receptors in stratum radiatum interneurones, resulting in increased levels of depolarisation and of GABA‐mediated inhibitory post‐synaptic potentials on CA1 neurons. In contrast, there was no change in NMDA sensitivity. *In vivo*, however, the loss of KAT‐2 would be relatively non‐specific since there may be several consequences of deleting such a major transaminase enzyme. KAT‐2 is known to accept a wide range of amino acids as substrates including α‐amino‐adipic acid which gives the enzyme its alternative name of α‐amino‐adipate transaminase. However, the authors consider only the possibility that the increase in α7‐nicotinic receptor activity following KAT‐2 deletion ‘can be accounted for’ purely by a loss of inhibition by endogenous kynurenate (Alkondon *et al.* 2004). While this result would be consistent with the authors’ proposal that kynurenate levels might normally maintain a degree of tonic suppression of nicotinic receptor sensitivity, it is only a supposition and does not represent positive evidence. The normality of NMDA sensitivity might be an adaptation to the loss of kynurenate as an endogenous inhibitor, with the increased nicotinic receptor sensitivity being the result of a series of events in the high complexity of CNS networks. This potential complexity is exemplified and supported by work showing that nicotine treatment and withdrawal results in altered glutamatergic function but no change of nicotinic receptor expression (Pistillo *et al.* 2016) and studies showing that nicotinic receptor activation alters the expression of NMDA receptors including those on the behaviourally important dopaminergic neurons (Salamone *et al.* 2014).

### Technical issues

In the light of this apparent lack of reproducibility, Albuquerque and Schwarcz (2013) presented a catalogue of technical differences in the methods employed in these various studies of kynurenic acid and nicotinic receptors. These included differences in the types of micropipettes and application systems used and the nature of the test preparation (such as slices or cultures) several of which had been taken into account experimentally by others noted above and some were incorrectly attributed to the use of oocytes rather than two human cell lines – GH4 cells expressing α7‐nicotinic receptors, and HEK293‐MRSII cells expressing transfected NMDAR subunits (Mok *et al.* 2009).

Overall, it is unlikely that the various details considered by Albuquerque and Schwarcz (2013) would have been consistently different between the work of Hilmas *et al. *(2001) and the four independent studies performed subsequently, using comparable concentrations and test preparations, to account for the variant results. It would be more rational to conclude that the negative results of the later studies are more likely to be correct in view of their consistency.

The most convincing explanation put forward by Mok *et al. *(2009) to explain any apparent blockade of nicotinic receptors was based on the ability of DMSO to reduce nicotinic responses. The highest concentration of kynurenic acid that they could achieve in DMSO was 100 mM, which would result in a final experimental concentration of 1% DMSO for a kynurenic acid concentration of 1 mM. Not only did this level reduce nicotinic receptor function, but different concentrations of kynurenate in the same final concentration of DMSO generated the same degree of inhibition, implying that it was the DMSO solvent that was the active component. While this explanation may be a major factor in the study by Hilmas *et al. *(2001), the same laboratory subsequently reported that the original results could be obtained using the more usual NaOH as the solvent (Alkondon *et al.* 2011). It may be relevant that normally inactive solubilizing materials such as Tween 80 and Triton X‐100 have been reported to block α7‐nicotinic receptors in a non‐competitive manner (Oz *et al.* 2004), perhaps suggesting an exceptionally broad susceptibility of these receptors to pharmacological interference and minor conformational perturbations.

## Interpreting results

While most of the foregoing discussion centres on direct attempts to replicate or explain results in vitro, a greater problem is encountered when considering studies in vitro or in vivo where experiments are performed under conditions where assumptions and interpretations of data are confounded by the complexity of neural connectivity and network.

### Transmitter release

For example, nicotinic receptors are present on many neurons and glial cells (Marchi *et al.* 2015). The two major subtypes of CNS nicotinic receptors, α7 and α4β2 are expressed on cell bodies and synaptic terminals (Wonnacot, 1997; Parikh *et al.* 2008, 2010; Puddifoot *et al.* 2015; Howe *et al.* 2016) where they promote the influx of extracellular calcium leading to the release of neurotransmitters (Turner 2004; Zappettini *et al.* 2010, 2014). Nicotinic receptors can regulate the release of, at least, glutamate (McGehee *et al.,* 1995; Gray *et al.,* 1996; Albuquerque *et al.* 2009; Gu *et al.,* 2012; Puddifoot *et al.* 2015; Pistillo *et al.* 2015, 2016; Howe *et al.* 2016; Ryu *et al.* 2017) and can modulate the release of GABA (Gray *et al.,* 1996; Le Magueresse *et al.,* 2006), dopamine (Schilstrom *et al.,* 1998a,b; Kaiser and Wonnacott 2000; Livingstone and Wonnacott, 2009; Livingstone *et al.* 2009) and norepinephrine (Pittaluga and Raiteri 1992; Pittaluga *et al.* 1992; Raiteri *et al.* 1992; Li *et al.,* 1998). It is reasonable to expect that the release of many other transmitters and modulators including serotonin, glycine, neuroactive peptides and perhaps kynurenines, proteases, cytokines and other factors may also be affected by nicotinic receptors on glia or synaptic terminals or cell bodies. This is a very significant problem of interpretation since not only would the effect of nicotinic receptor stimulation then be indirect, but the endogenous compounds whose release is affected would in turn affect neuronal excitability and transmitter release, generating the network complexity which always bedevils work in the CNS. Several groups have specifically noted that the effects of nicotinic receptor activation on transmitter release are not direct, but mediated indirectly via the release of glutamate and the activation of NMDA receptors (Schilstrom *et al.* 1998a,b). Many of the NMDA receptors on nerve terminals are blocked by kynurenic acid including those which can release acetylcholine (Bouvier *et al.* 2015, 2018; Banerjee *et al.* 2016) and other transmitters including glutamate (Ransom and Deschenes, 1989; Fink *et al.,* 1990; Krebs *et al.,* 1991; Overton and Clark, 1991; Garcia‐Munoz *et al.,* 1991).

### In vitro pharmacology

A major complication in the interpretation of results is that α7 receptor activation is usually facilitated by a degree of glutamate receptor activation (Gray *et al.* 1996; Fu *et al.* 2000; Hilmas *et al.* 2001). Indeed a report by Alkondon *et al. *(2003) concluded that the activation of NMDA receptors and AMPA receptors contributed directly to the modulation of cholinergic excitation. This strongly supports the above arguments that the apparent loss of α7 sensitivity by kynurenate is likely to be due to the well‐established blockade of glutamate receptors with secondary, indirect effects on nicotinic receptors. Blockade by kynurenic acid of the facilitatory glutamate sites would remove their facilitation of nicotinic receptor activity and generate an apparent reduction in cholinergic sensitivity.

A further complication of nicotinic receptors is the functional and histochemical evidence for their frequent co‐expression with other receptors (Vizi and Lendvai, 1999; Sher *et al.,* 2004; Patti *et al.,* 2006; Grilli *et al.,* 2008, 2009a,b; Lin *et al.,* 2010; Marchi and Grilli, 2010; Salamone *et al.* 2014), including receptors for NMDA and AMPA (Marchi *et al.,* 2009, 2015; Marchi and Grilli, 2010), the combined activity of which can modify synaptic function and transmitter release (Raiteri *et al.,* 1992; Musante *et al.,* 2011). Complexes usually imply crosstalk between the component receptors such as the ability of nicotinic receptor to up‐ or down‐regulate the expression of glutamate receptors (Pistillo 2015, 2016) and may account for the report that activation of α7nicotinic receptors is required for the activation of NMDA receptors in the prefrontal cortex (Yang *et al.* 2013).

### In vivo pharmacology

A number of studies have attempted to understand the effects of kynurenic acid by examining its pharmacological interactions with other neuroactive compounds *in vivo*. The interpretation of kynurenate pharmacology is most contentious in behavioural studies in which there are two areas of particular concern: (i) the use of derivatives of kynurenic acid which are assumed, incorrectly, to have the same sites and mechanisms of action as kynurenate itself (ii) the use of galantamine, on the assumption that it acts uniquely on nicotinic receptors and is therefore diagnostic of their involvement, but whose basic mechanism of action cannot be confirmed and is in doubt (Kowal *et al.* 2018; see below).

### Kynurenic acid derivatives

Following the discovery that the activation of glutamate receptors by NMDA required the allosteric co‐agonist glycine (Johnson and Ascher 1987), it was recognised that antagonists of NMDA could act directly on the glutamate/ NMDA binding site (Olverman *et al.* 1988; Moroni et al., Danysz et al., Robinson et al.) or the strychnine‐resistant glycine co‐agonist site. Indeed it is now clear that kynurenic acid can act directly on both of these sites (Stone *et al.* 2013). Detailed biophysical analyses have indicated that kynurenate is a competitive antagonist at the glutamate/NMDA recognition site (Mayer *et al.* 1988; Birch *et al.,* 1988a,b; Pullan and Cler 1989; Kloog *et al* 1990) but also blocks NMDA at the NMDA co‐agonist glycine‐2 site partly by a non‐competitive inhibition, although there has been debate on the relative competitive or non‐competitive nature of this activity (Mayer *et al.* 1988; Watson *et al.,* 1988; Birch *et al.* 1988a,b; Dingledine *et al.* 1990; Henderson *et al.* 1990) although increasing glycine concentrations can displace kynurenate and reverse the blockade (Pullan and Cler 1989; Kessler *et al.,* 1989a,b; Danysz *et al.* 1989a,b). It should be stressed that many of these detailed single‐cell analyses of kynurenate pharmacology and receptor kinetics were based on results from mammalian hippocampal neurons and repeatedly confirmed the blockade of NMDA receptors by kynurenate.

Later structure‐activity studies have identified derivatives of kynurenate with higher potency and greater selectivity as antagonists at these sites (Leeson *et al.* 1991; Leeson and Iversen, 1994; Rover *et al.* 1997; Stone 2000a,b, 2001b). Prominent among these are compounds which, by acting almost exclusively on the strychnine‐resistant glycine site, should show greater suppression of NMDA receptor activation relative to AMPA or kainate receptors, at which kynurenic acid has lower potency (Fisher and Mott, 2011).

From this work the NMDAR antagonists most often used experimentally are 7‐chloro‐kynurenic acid (7CKA) or 5,7‐dichloro‐kynurenic acid (57diCKA) but it must be emphasised that these do not act at exactly the same sites as kynurenic acid. Both 7CKA and 57diCKA are highly selective antagonists acting only on the glycine co‐agonist site of the prominent NR1/2 subunits of NMDA receptors (Kemp *et al.* 1988; Kleckner and Dingledine, 1989; Dingledine *et al.,* 1990; Benveniste *et al.* 1990; Benveniste and Mayer, 1991; Priestley *et al.* 1995). Thus, any action of kynurenate at the glutamate or NMDA recognition site will ***not*** be reproduced by 7CKA or 57diCKA. The argument that an action of kynurenate which is not mimicked by 7CKA or 57diCKA must therefore be mediated by a nicotinic (or other) receptor is therefore completely spurious.

Furthermore, chemical analogues or derivatives, even with extremely minor structural modifications, are distinct molecules with individual profiles of activity and physico‐chemical properties. Not only do 7CKA and 57diCKA exhibit different potencies and selectivities of action at glycine binding sites compared with kynurenate, but their absolute and relative activities depend on the cell type, location, activity, chemical microenvironment and, most importantly, subunit composition of the receptors (Nilsson *et al.* 2007; Smothers and Woodward 2007). In some situations 7CKA *increases* the binding of the competitive NMDA receptor antagonist [H‐3]CGP39653 (Oblin and Schoemaker, 1994). The behaviour of 7CKA and glycine are different at the glycine sites, since the modulatory polyamine spermine enhances glycine binding but not that of 7CKA, possibly implying a different molecular binding pattern at the receptor (Marvizon and Baudry, 1994).

Linderholm *et al. *(2007) obtained results which strongly suggest that kynurenic acid does *not* act on α7‐nicotinic receptors. This group reported that 4‐chlorokynurenine, which crosses the blood‐brain barrier and is converted to 7CKA in the CNS, *increased* the excitability of neurons in the ventral tegmentum. The same result was obtained using the selective NMDA receptor blocker SDZ‐220‐581 but not the α7‐nicotinic receptor blocker MLA, indicating that the increase in tegmental firing rates were due to 7CKA acting as an antagonist at glutamate receptors but *not* α7‐nicotinic receptors. In this case, 7CKA was probably acting at the same site as kynurenic acid since increasing endogenous cerebral kynurenate concentrations by kynurenine administration produced a similar increase in tegmental activity. A similar selectivity for NMDA rather than nicotinic sites *in vivo* was also reported following intrathecal administration of kynurenic acid (Tuboly *et al.* 2015).

### Subunit composition

The important issue of subunit structure and receptor composition is illustrated by the actions of these derivatives on receptors which include NR3 subunits where it was shown that 7CKA does *not* displace glycine from combinations of NR1 with NR3A or NR3B subunits (Nilsson *et al.* 2007; Smothers and Woodward, 2007) and does not antagonise their activation. This contrasts with the blockade of NR1/NR2 subunit combinations. Indeed, 7CKA has been reported to *potentiate* activation of NR3 subunits in studies where 57diCKA was an antagonist (Smothers and Woodward, 2007). This difference between 7CKA and 57diCKA probably reflects earlier conclusions that the two compounds act, to some extent, at different sites (Baron *et al.* 1990, 1991). Clearly, the net effects of kynurenic acid, 7CKA and 57diCKA will depend on the density and location of different NMDAR subunit combinations and, most importantly, the ratios between their respective levels of expression, activation and sensitivity.

Additional problems may apply in the complexity of *in vivo* studies, since 7CKA may appear to block non‐NMDA as well as NMDA receptors and may do both in a non‐competitive manner which is not reversed by glycine site agonists (Lehmann *et al.* 1993), results which have not been noted in isolated cells and tissues. In some cases the different ratios of activity of compounds on the glutamate recognition site compared with the NMDA‐glycine site have been correlated with major differences in their behavioural profiles (Grimwood *et al.* 1995; Kehne *et al.* 1995).

There have also been repeated indications that the NMDA co‐agonist glycine binding sites may exist in several forms, differentially affected by kynurenic acid, 7CKA or 57diCKA (Danysz *et al.* 1989a,b; Yoneda *et al.,* 1994). The stated conclusion of one such study was that “These data support the possibility that different glycine receptor antagonists may have different therapeutic targets” (Kehne *et al.* 1995) emphasising that kynurenic acid, 7CK and 57diCKA cannot be considered pharmacologically equivalent. This conclusion is strongly supported by evidence that there may be several binding sites for glycine on NMDA receptors since there are clear regional differences in the stoichiometry of glycine and NMDA interactions (O’Shea *et al.* 1991).

There are, therefore, many factors operating *in vitro* and, especially, *in vivo*, which mean that the sites of action of kynurenic acid, 7CKA and 57diCKA are different and determined by the concentration of the compounds themselves at key molecular sites, the concentrations of endogenous ligands (glutamate, glycine, D‐serine) and the subunit composition of the NMDARs among many other factors. No meaningful conclusions can therefore be drawn from the use of these compounds in identifying molecular sites of action as being on NMDA or non‐NMDA receptors, especially nicotinic receptors.

## Galantamine as a pharmacological tool

Galantamine is one of several compounds originally identified as acetylcholinesterase inhibitors for use in disorders such as Alzheimer's disease. Subsequent reports have claimed that the drug was also an Allosteric Potentiating https://en.wikipedia.org/wiki/Ligand_(biochemistry) at nicotinic receptors with several subunit compositions (Maelicke *et al.* 2001; Samochocki *et al.* 2003; Albuquerque *et al.* 2009; Liu *et al* 2010). This putative mechanism of action has been used to support the hypothesis that kynurenic acid can block α7‐nicotinic receptors since, it is argued, if an effect of kynurenate can be prevented or reversed by galantamine, kynurenate must be acting by blocking α7‐nicotinic receptors – the activity of kynurenate is assumed to have been countered by the abilities of galantamine to produce an allosteric potentiation of α7‐nicotinic receptor‐mediated responses (in addition to its acetylcholinesterase activity). However, there are several reasons why this argument is not tenable, especially since recent work by Kowal *et al. *(2018; discussed below) has failed to demonstrate any allosteric potentiating activity of galantamine at nicotinic receptors (Roman et al. 2005).

In particular, the most that can be said is that the activity of galantamine opposes (rather than reverses) the action of kynurenate, a conclusion which mirrors the comments (expanded below) that the interaction is not of true antagonism, but of ‘response cancellation’ – galantamine simply had the opposite effect to kynurenic acid, increasing the size of the IPSCs and apparently preventing the inhibitory action of kynurenate.

As an example, raising the levels of kynurenic acid in the cerebral cortex by administering kynurenine was associated with deficits in cognitive performance tasks such as attentional set‐shifting (Alexander *et al.,* 2012, 2013). Control levels of performance were restored when animals were treated with galantamine, a result taken to indicate that kynurenic acid had been working as an α7‐nicotinic receptor antagonist. However, such a conclusion is entirely inappropriate as discussed below. In related work, Pershing *et al., *(2015) employed partial agonists at ɑ7‐nicotinic receptors to demonstrate that they could successfully overcome behavioral impairments caused by kynurenate, but making a similar incorrect inference that this indicated that kynurenate was acting at α7‐nicotinic receptors.

These are just two examples of where a fundamental principle of pharmacology has not been recognised. A compound X may appear to be an antagonist of Y if it merely exhibits the opposite activity, thus cancelling the action of X but with no interaction at the receptor sites or transduction pathways. The example usually used to illustrate this concept of ‘physiological antagonism’ or ‘response cancellation’ is that of autonomic regulation in the heart. Acetylcholine lowers heart rate and the addition of adrenaline negates this effect, while adrenaline increases heart rate but this is prevented by acetylcholine. However, neither of these compounds is an antagonist of the other in the pharmacological meaning of the term, and they clearly act on completely different sets of receptor. The entire basis of pharmacology and drug development is based on well‐established and recognised concepts of ‘receptors’ and their agonists and antagonists, and response cancellation must always be *eliminated* as an explanation of results, not used to draw meaningless conclusions about drug targets, receptors or sites of action. Results from such work cannot be used as positive or confirmatory evidence for a receptor‐based hypothesis, nor does it exclude a myriad of alternative possible explanations, some of which are far more likely to be correct. The logical error involved is equivalent to concluding that a simple correlation between two factors can be taken to indicate cause and effect. In this particular case the most probable explanation of the results – and possibly many similar observations by others – is that galantamine and the α7‐nicotinic receptor agonists were acting on molecular targets or neurons with actions interfering with those affected by kynurenic acid.

The ‘galantamine test’ and kynurenate analogues discussed above (7CKA and 57diCKA) have also been used in neurochemical studies of GABA concentrations in the rat striatum (Beggiato *et al.* 2013) or prefrontal cortex (Beggiato *et al.* 2014). Infusions of kynurenic acid reduced the extracellular concentration of GABA, an effect ascribed to nicotinic receptor blockade on the grounds that (a) the effect was reversed by galantamine and (b) the effect was not replicated by 7CKA. As noted above this type of result merely indicates the opposing, but not necessarily antagonistic, actions of kynurenate and galantamine. Similarly, the absence of any effect of 7CKA was interpreted to exclude NMDAR involvement on the basis that 7CKA and kynurenate should have the same activity on NMDARs, an interpretation which, as noted above, is incorrect.

In a related study kynurenic acid reduced the concentrations of glutamate in the cerebral cortex (Konradsson‐Geuken *et al.* 2010; Wu *et al.* 2010) and the reversal of this by galantamine was claimed to indicate that kynurenic acid blocks the galantamine‐sensitive allosteric potentiation site on α7‐nicotinic receptors. Similarly, kynurenate suppressed dopamine release in the rat striatum and the effect was prevented by galantamine leading to the conclusion that kynurenate was acting on α7‐nicotinic receptors (Wu *et al.* 2007; Rassoulpour *et al.* 2005). In reality, both kynurenic acid and galantamine could have been acting on any of their totally independent confirmed target sites to produce opposite effects on glutamate levels: the results offered no direct (or indirect) support to indicate an action specifically on nicotinic receptors for either compound. The results are at best only suggestive or consistent with the authors’ concept and with many others which cannot be excluded. This criticism is strengthened by reports that galantamine alone increases dopamine release by acting on α7‐nicotinic receptors (Wang *et al.* 2007), so that a ‘cancellation’ of an inhibitory effect of kynurenate by a positive action of galantamine may again be involved rather than a true antagonist action.

In marked contrast to the above studies, several groups have performed similar work without using galantamine and have reached conclusions that the effects of kynurenic acid on dopaminergic projections to the Ventral Tegmental Area can be explained entirely in terms of its blockade of NMDA receptors without any need to invoke the involvement of acetylcholine, nicotinic receptors or any other mechanism which does not involve NMDA receptors. As noted above, Linderholm *et al. *(2007) demonstrated that 7CKA or a selective NMDA antagonist increased neural excitability although the α7‐nicotinic receptor blocker MLA did not leading to the conclusion that the excitation was due to 7CKA blocking glutamate receptors but *not* α7‐nicotinic receptors.

### Galantamine and NMDA receptors

Conclusions using galantamine in particular are often not justified because the compound itself has a complex pharmacology. First, although it does block acetylcholinesterase, it also inhibits other esterase enzymes (Nordberg and Svensson, 1998; Darvesh *et al.* 2003), some of which hydrolyse neuroactive peptides including the neurokinins and may affect other proteinaceous compounds affecting excitability such as growth factors and cytokines.

Second, galantamine has several recognised sites of action other than nicotinic receptors, including the ability to potentiate response to NMDA receptor activation (Moriguchi *et al* 2004; Narahashi *et al.* 2004; Zhao *et al.* 2006). Although the authors subsequently suggested that this effect was mediated indirectly via nicotinic receptors (Moriguchi *et al.* 2009a), their earlier experiments seem to have been conducted in the absence of an NMDA receptor blocker, since 2‐amino‐5‐phosphono‐pentanoic acid was used only to confirm the NMDAR‐dependency of long‐term potentiation (LTP) but was not included in the experiments on galantamine. Thus, many of the observations could have been mediated indirectly via NMDA receptor activity and enhancement resulting from induced glutamate release, whether or not there was a contribution from α7‐nicotinic receptors, and the conclusions are not secure. On balance these studies remain consistent with the original explanation (Moriguchi *et al.* 2004; Narahashi *et al.* 2004; Zhao *et al.* 2006) that galantamine can enhance NMDAR activation.

Many studies have reported effects of galantamine on synaptic transmission involving glutamate receptor activation. α7‐nicotinic receptors facilitate glutamatergic neurotransmission and LTP in the hippocampus (Radcliffe and Dani 1998; Mansvelder and McGehee 2000; Gu *et al.* 2012; Puddifoot *et al* 2015) and other regions such as the amygdala (Jiang *et al.* 2016). Galantamine potentiates NMDA‐induced depolarisation as noted above and the effects of galantamine can be blocked by NMDAR antagonists (Schilstrom *et al.* 2007). Clearly, these agonist actions of galantamine on NMDARs would explain its ability to reverse the behavioural effects of NMDA receptor inhibitors such as dizocilpine (MK‐801) as well as the effects of kynurenic acid without involving nicotinic receptors at all. Recent reports that a combination of galantamine and memantine show improved cognition‐enhancing activity compared to either alone (Koola 2018) are compatible with these data since galantamine could increase the beneficial effects of memantine on the neuronal signal‐to‐noise ratio by potentiating NMDA receptor activation without the need to invoke nicotinic receptor involvement.

For example, galantamine increases the magnitude of LTP in the hippocampus (Moriguchi *et al.* 2009b; Forrest *et al.* 2015) by a mechanism which involves calcium/calmodulin‐dependent protein kinase II and activation of Protein Kinase C. An involvement of nicotinic receptors was concluded from the ability of α‐bungarotoxin to block the galantamine activity on α7‐nicotinic receptors, whereas dihydro‐β‐erythroidine (DHβE), blocking mainly α4β2‐nicotinic receptors, had no effect. The authors suggested that an initial enhancement of α7‐nicotinic receptors could account for the succeeding activation of protein kinases and NMDARs. However, the data do not exclude additional indirect effects of galantamine on neurons affecting LTP, or direct effects potentiating NMDAR activation.

Nicotinic receptors exist on glutamatergic synaptic terminals and can promote glutamate release (McGehee *et al.,* 1995; Gray *et al.,* 1996; Albuquerque *et al.,* 1997; Wonnacott, 1997; Alkondon, Pereira, Barbosa *et al.,* 1997; Alkondon, Pereira, Cortes *et al.,* 1997; Aramakis and Metherate, 1998; Radcliffe and Dani, 1998; Mansvelder and McGehee, 2000) and enhance LTP (Santos *et al.* 2002; Ge and Dani 2005). However, even when produced by a selective agonist at α7nicotinic receptors, an action blocked by MLA or α7‐nicotinic receptor deletion, galantamine did not modify the induced LTP (Lagostena *et al* 2008). These results might imply that galantamine can facilitate NMDA receptor function in inducing LTP in the absence of nicotinic receptor activity. To examine this possibility, the effect of galantamine was studied on NMDA‐dependent LTP in hippocampal slices incubated with the nicotinic receptor blockers MLA and DHβE (Forrest *et al.* 2015). The results showed that galantamine does indeed potentiate NMDA‐dependent LTP induced by a theta‐stimulation protocol, confirming that the drug could reverse or antagonise an effect produced by blocking NMDA receptors in the presence of nicotinic receptor blockade. This is consistent with the earlier reports that galantamine can facilitate LTP (Moriguchi *et al.* 2009b) but in the absence of nicotinic receptor activation the results support the proposal that the effect is via the activation or modulation of NMDARs. Interestingly, Alkondon *et al. *(2003) had earlier published that NMDA receptors might contribute to the effects of α7‐nicotinic receptor agonists, a finding which, if expanded, might explain many of the controversial data discussed above.

The problems of using galantamine are not confined to the interpretation of kynurenic acid activity. Dizocilpine is another well‐established antagonist at NMDA receptors and its administration to rats produces deficits in learning and memory. These effects were prevented by the co‐administration of galantamine (Su *et al.* 2014) but, while this would be most readily explained by the well documented opposing *but independent* actions of dizocilpine and galantamine on NMDA receptors as summarised above, the authors interpreted the results to imply that dizocilpine was working via changes in α7‐nicotinic receptor function. In this case, the far more obvious, simple and well‐established explanation has been ignored in favour of a more complex one on the assumption that galantamine is an entirely specific compound with a single pharmacological action to promote cholinergic function. The recognised activity of dizocilpine at NMDARs interacting with the less‐established action of galantamine on NMDARs has been ignored.

In related work, galantamine reversed the suppression of pre‐pulse inhibition produced by dizocilpine in a model for schizophrenia (Shao *et al.* 2014) and this interaction was also interpreted as implicating α7‐nicotinic receptors in the phenomenon with no consideration of the major established alternative explanations of NMDA receptor involvement, despite the fact that dizocilpine is a recognised, highly selective antagonist at NMDA receptors.

A similar story applies to another NMDA antagonist, phencyclidine. McLean *et al *(2011) examined the effects of phencyclidine on a range of cognitive functions in rats. Phencyclidine blocks the NMDAR‐associated ion channels in a manner similar if not identical to dizocilpine and the effects of phencyclidine, therefore, are *unambiguously* attributable to NMDAR blockade. However, the activation of α7‐nicotinic receptors by a selective full agonist prevented and reversed these effects. Thus, the principle that α7‐nicotinic receptor activation necessarily indicates an involvement of α7‐nicotinic receptors in the mechanism of action of a test compound (such as kynurenic acid or 7CKA) cannot be assumed or supported in this way.

Some groups have recognised these limitations of interpretation and have attempted to strengthen their interpretation of the actions of kynurenate by showing that α7‐nicotinic receptor blockers can prevent the effects of galantamine overcoming the actions of kynurenate. However, such observations and arguments remain entirely irrelevant as they do not exclude the possibility that galantamine might be producing its effects by acting at NMDA receptors on neurons which then secondarily activate α7‐nicotinic receptors to yield the final result.

The same principles apply to many combinations of agonist and antagonist ligands examined on neuronal networks. One relevant and cautionary study showed that the impairment of memory by scopolamine, a well‐established antagonist at muscarinic receptors, was prevented by the nicotinic antagonist mecamylamine (Newman and Gold 2016). That does not, of course, imply that scopolamine is acting on nicotinic receptors, only that α7‐nicotinic receptors are involved somewhere along the neuronal circuitry involved in memory processing even when they also involve NMDA receptors (Yang *et al.* 2013). Interestingly, galantamine has been reported to act partly on muscarinic receptor subtypes in some situations (Almasieh *et al.* 2010). Indeed, Wadenberg *et al. *(2012) have shown that the potentiation by galantamine of antipsychotic drug efficacy in conditioned avoidance testing is prevented by muscarinic but not nicotinic receptor blockers, leading to their proposal that anti‐psychotic activity of galantamine involves mainly muscarinic receptors. This would be supported by evidence that the abnormal pre‐pulse inhibition seen in mice reared in isolation is normalised by a muscarinic action of galantamine (Yano *et al.* 2009) and is not affected by a different acetylcholinesterase inhibitor such as donepezil (Koda *et al.* 2008). Since the various cholinesterase inhibitors have different structures, they are each likely to have a spectrum of minor effects on several targets, giving them their distinctive pharmacological and clinical profiles.

### The broad pharmacology of galantamine

At this point it is appropriate to emphasise that galantamine does have a very complex pharmacology in addition to the actions on NMDA receptors and transmitter release discussed above (Ago *et al.* 2011). In particular, galantamine can facilitate or inhibit glutamate release (Santos *et al.* 2002; Nagumo *et al.* 2011; Ondrejcak *et al.* 2012; Cheng and Yakel 2014). While that result could depend on neuronal activity which could be excluded by the presence of tetrodotoxin, it is clear that many nicotinic receptors, including α7‐homomeric receptors, are present on synaptic terminals (Cheng and Yakel 2014). The induction or facilitation of glutamate release at these presynaptic sites would not be affected by tetrodotoxin and would be difficult to exclude from contributing to a pharmacological response, unless a functionally complete genetic deletion could be achieved. The overall effect of altered glutamate release would be to produce an apparent up‐ or down‐regulation of excitability respectively with a resulting modulation of neurochemical and behavioural changes depending on the neuronal population and CNS region being investigated.

A significant concern regarding many of the conclusions drawn with galantamine is that this compound also blocks several types of potassium channels (Pan *et al.* 2003; Zhang *et al.* 2004; Vicente *et al.* 2010; Vigneault *et al.* 2012). Since kynurenic acid tends to reduce neuronal excitability, irrespective of whether it is blocking NMDA, AMPA or kainate receptors, the depolarisation induced by blocking potassium conductances will appear to reverse those effects – another example of ‘physiological antagonism’ or ‘signal cancellation’ (see above). This effect is sufficiently potent to contribute to the pro‐cognitive actions of galantamine (Vicente *et al.* 2010).

Third, at a concentration of around 1μM galantamine can block synaptic terminal calcium‐dependent potassium channels (Ales *et al* 2006) probably contributing to its promotion of transmitter release. At higher levels galantamine also suppresses spike after‐hyperpolarization and neuronal accommodation in the hippocampus (Oh *et al.* 2006). Interestingly these effects were mediated through muscarinic receptors, not nicotinic receptors, and the authors noted that no enhancement of EPSPs could be detected unless excitability was increased by the inclusion of a GABA‐A receptor blocker.

Fourth, and perhaps most instructively, galantamine can even interfere with the release and actions of protein mediators such as Insulin‐like Growth Factor‐2 (IGF2), Fibroblast Growth Factor‐2 (FGF2) and Brain‐Derived Neurotrophic Factor (BDNF) in the hippocampus (Kita *et al.* 2013). Galantamine also reduces significantly the production and release of Tumor Necrosis Factor‐α in rats with polysaccharide‐induced inflammation (Liu *et al.* 2010) and can suppress the induction of iNOS, resulting in lowered NO production during hypoxia (Egea *et al.* 2012). Galantamine is an efficient anti‐oxidant agent, preventing cell damage caused by hydrogen peroxide (Triana‐Vidal and Carvajal‐Varona 2013) or amyloid‐β (Melo *et al* 2009), this possibly being one of the mechanisms by which the drug protects against neuronal damage by β‐amyloid (Matharu *et al,* 2009; Li *et al.* 2010; Rao *et al.* 2013) and the toxic compounds kainic acid and caffeic acid (Kumar *et al.* 2011). There is, therefor, a plethora of actions which, independently or combined, provide galantamine with a complex pharmacology which make it even more inappropriate as a tool to define the pharmacological specificity of a compound such as kynurenic acid.

## Conclusions and Implications

This discussion has focused on a problem arising from data which have not been reproducible in at least 13 independent studies (Table 1), but which have spawned other studies which appear to be supportive but are actually only ‘consistent’ with those data and do not include any results which substantiate the concept of kynurenic acid blocking nicotinic receptors. The interpretation of results based on those studies is therefore insecure, but still sometimes ignores a wealth of contrary, established, data using dizocilpine [MK‐801], phencyclidine and other well‐characterized compounds. The problem is compounded by a reliance on chemical tools which are unsuitable for drawing definitive conclusions. Galantamine is not only a rather non‐selective drug but the widely accepted mechanism of action (allosteric potentiation) is itself not reproducible (Kowal et al. 2018). All these arguments are made even more difficult in the CNS with its plethora of sites available for drug effects at pre‐synaptic and post‐synaptic receptors, sub‐synaptic and extrasynaptic locations, many on complex networks of neurons that make interpretation extremely difficult and dependent on earlier and recent neuronal activity. Until a wider consensus is obtained based on more positive results and reliable pharmacological analyses, it is clearly inappropriate to refer to kynurenic acid as a ‘nicotinic receptor blocker’ especially without any qualification or reference to its more well‐established actions. Since it is one of the fundamental tenets of the Scientific Method that data should be reproducible, then the current default position must be that kynurenic acid does NOT directly affect nicotinic receptors and results obtained with it must be re‐assessed on the basis of its fully documented actions on NMDA, AMPA and kainate receptors, with the possible involvement of AHRs or GPR35. This is particularly so since the inability to block nicotinic receptors at the same time that glutamate receptors or synaptic potentials are blocked (viewed as a positive control) (Table 1) is highly reliable evidence against nicotinic blockade. It will be necessary for independent laboratories to examine recombinant proteins in isolation transfected into cells (e.g. chinese hamster ovary cells cells, HeLa cells, or oocytes) which do not constitutively express any of the relevant competing receptors and which are not networked as in the normal CNS. Unfortunately even those approaches will need to consider the effects of an absence of hormones, growth factors, neuroactive compounds, receptors, ecto‐enzymes, extracellular matrix components, hormones, trace elements etc. which may influence receptor structure and ligand binding. Most importantly, the use, analysis and discussion of results under any experimental conditions must be interpreted with due consideration of the network complexity – structural and chemical – which characterises the CNS, in addition to the correct understanding of pharmacological principles. In view of the increasing medical importance of the kynurenine pathway in a range of psychiatric, neurological and metabolic disorders, and its growing importance as a target for new drug development, it is essential that the sites of action and pharmacology of these tryptophan catabolites are clearly and fully understood.
